# Supplementary material for: Lipidomic profiling of human serum enables detection of pancreatic cancer
Source: Nat Commun. 2022 Jan 10;13:124. doi: 10.1038/s41467-021-27765-9 (PMC8748654; doi:10.1038/s41467-021-27765-9)
Supplement: Supplementary file 1 — Supplementary Information [file 41467_2021_27765_MOESM1_ESM.pdf]

SUPPLEMENTARY INFORMATION

Lipidomic profiling of human serum enables detection of pancreatic cancer

Denise Wolrab[1], Robert Jirásko[1], Eva Cífková[1], Marcus Höring[2], Ding Mei[3,4], Michaela Chocholoušková[1], Ondřej Peterka[1], Jakub Idkowiak[1], Tereza Hrnčiarová[1], Ladislav Kuchař[5], Robert Ahrends[6], Radana Brumarová[7], David Friedecký[7], Gabriel Vivo-Truyols[8], Pavel Škrha[9], Jan Škrha[10], Radek Kučera[11], Bohuslav Melichar[12], Gerhard Liebisch[2], Ralph Burkhardt[2], Markus R. Wenk[3,4], Amaury Cazenave-Gassiot[3,4], Petr Karásek[13], Ivo Novotný[13], Kristína Greplová[14,15], Roman Hrstka[15], Michal Holčapek[1*]

[1] Department of Analytical Chemistry, Faculty of Chemical Technology, University of Pardubice, Pardubice, Czech Republic.

[2] Institute of Clinical Chemistry and Laboratory Medicine, University Hospital of Regensburg, Regensburg, Germany.

[3] Singapore Lipidomics Incubator (SLING), Life Sciences Institute, National University of Singapore, Singapore.

[4] Department of Biochemistry, Yong Loo Lin School of Medicine, National University of Singapore, Singapore.

[5] Research Unit for Rare Diseases, Department of Pediatrics and Inherited Metabolic Disorders, First Faculty of Medicine, Charles University and General University Hospital in Prague, Prague, Czech Republic.

[6] Department of Analytical Chemistry, University of Vienna, Vienna, Austria.

[7] Palacký University Olomouc, Institute of Molecular and Translational Medicine, Olomouc, Czech Republic.

[8] Tecnometrix, Ciutadella De Menorca, Spain.

[9] Third Faculty of Medicine, Charles University, Prague, Czech Republic.

[10] 3rd Department of Internal Medicine, First Faculty of Medicine, Charles University, Czech Republic.

[11] Department of Immunochemistry Diagnostics, University Hospital in Pilsen, Pilsen, Czech Republic.

[12] Department of Oncology, Faculty of Medicine and Dentistry, Palacký University and University Hospital, Olomouc, Czech Republic.

[13] Clinic of Comprehensive Cancer Care, Masaryk Memorial Cancer Institute, Brno, Czech Republic.

[14] Faculty of Medicine, Masaryk University, Brno, Czech Republic.

[15] Research Centre for Applied Molecular Oncology, Masaryk Memorial Cancer Institute, Brno, Czech Republic.

* Correspondence to: Michal.Holcapek@upce.cz

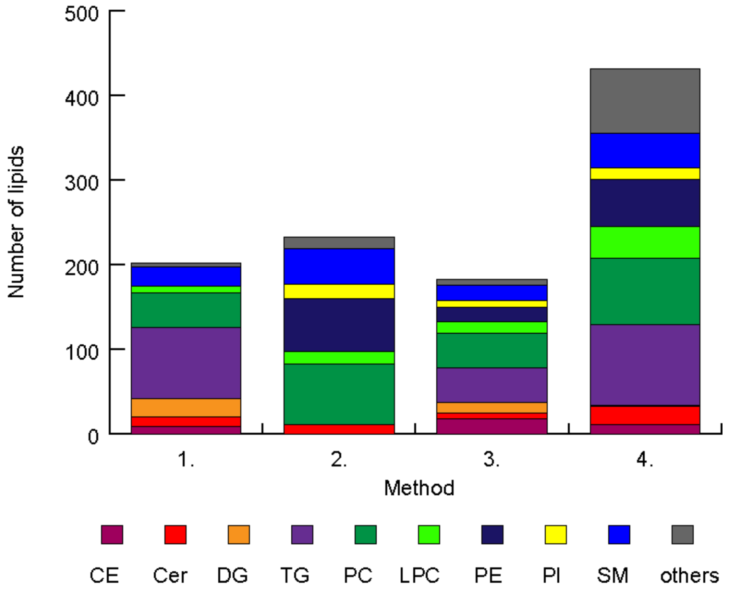

Supplementary Fig. 1 Summary of quantified lipid species for particular lipid classes. Method 1 – UHPSFC/MS measured by lab 1 (n=202), Method 2 – shotgun MS with low-resolution (LR) measured by lab 2 (n=232), Method 3 – shotgun MS with high-resolution (HR) measured by lab 2 (n=183), and Method 4 – RP-UHPLC/MS measured by lab 3 (n=431). Annotation of lipid classes: CE – cholesteryl esters, Cer – ceramides, DG – diacylglycerols, TG – triacylglycerols, PC – phosphatidylcholines, LPC – lysophosphatidylcholines, PE – phosphatidylethanolamines, PI – phosphatidylinositols, and SM – sphingomyelins.

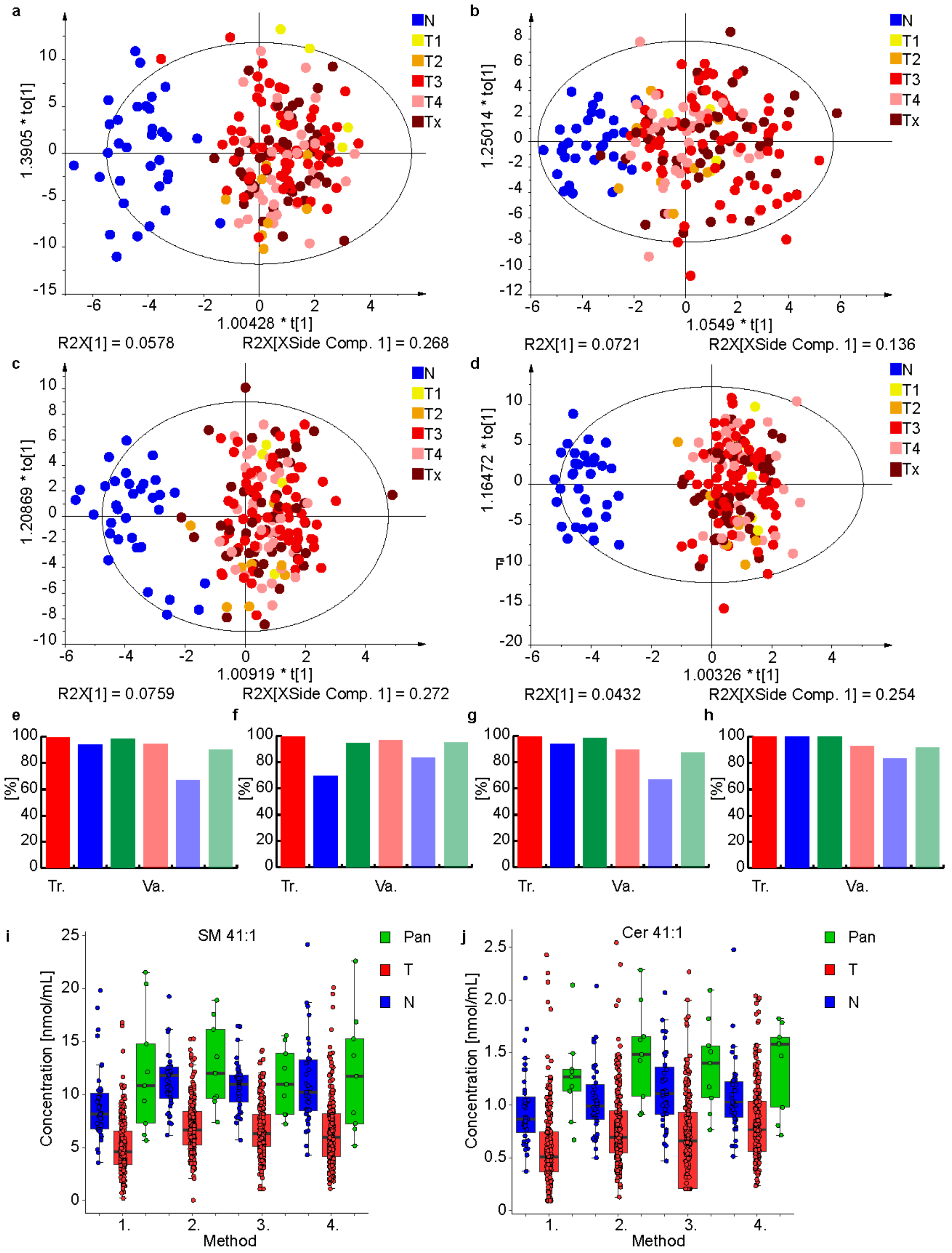

Supplementary Fig. 2 Results for the lipidomic profiling of male serum samples from PDAC patients and healthy controls in the Phase II. OPLS-DA for molar concentrations of lipid species obtained for the training set: **a** UHPSFC/MS (166 T + 33 N), **b** shotgun MS (LR)

(165 T + 33 N), **c** shotgun MS (HR) (164 T + 33 N), and **d** RP-UHPLC/MS (166 T + 33 N). Individual samples are colored according to their tumor (T) stage: T1 – yellow, T2 – orange, T3 – red, T4 – rose, Tx – brown (information about the stage is not available). Sensitivity (red), specificity (blue), and accuracy (green) values in percentage for the training (Tr.) and validation (Va.) sets: **e** UHPSFC/MS, **f** shotgun MS (LR), **g** shotgun MS (HR), and **h** RP-UHPLC/MS. Box plots of molar lipid concentrations normalized with the NIST reference material determined in PDAC patients (222 T), controls (39 N), and pancreatitis (9 Pan) patients including both validation and training sets for **i** SM 41:1 and **j** Cer 41:1 using UHPSFC/MS (Method 1), shotgun MS (LR) (Method 2), shotgun MS (HR) (Method 3), and RP-UHPLC/MS (Method 4). In each box plot, the centerline represents the median, the bounds represent the 1st and 3rd quartile and whiskers span 1.5 fold inter-quartile range from the median.

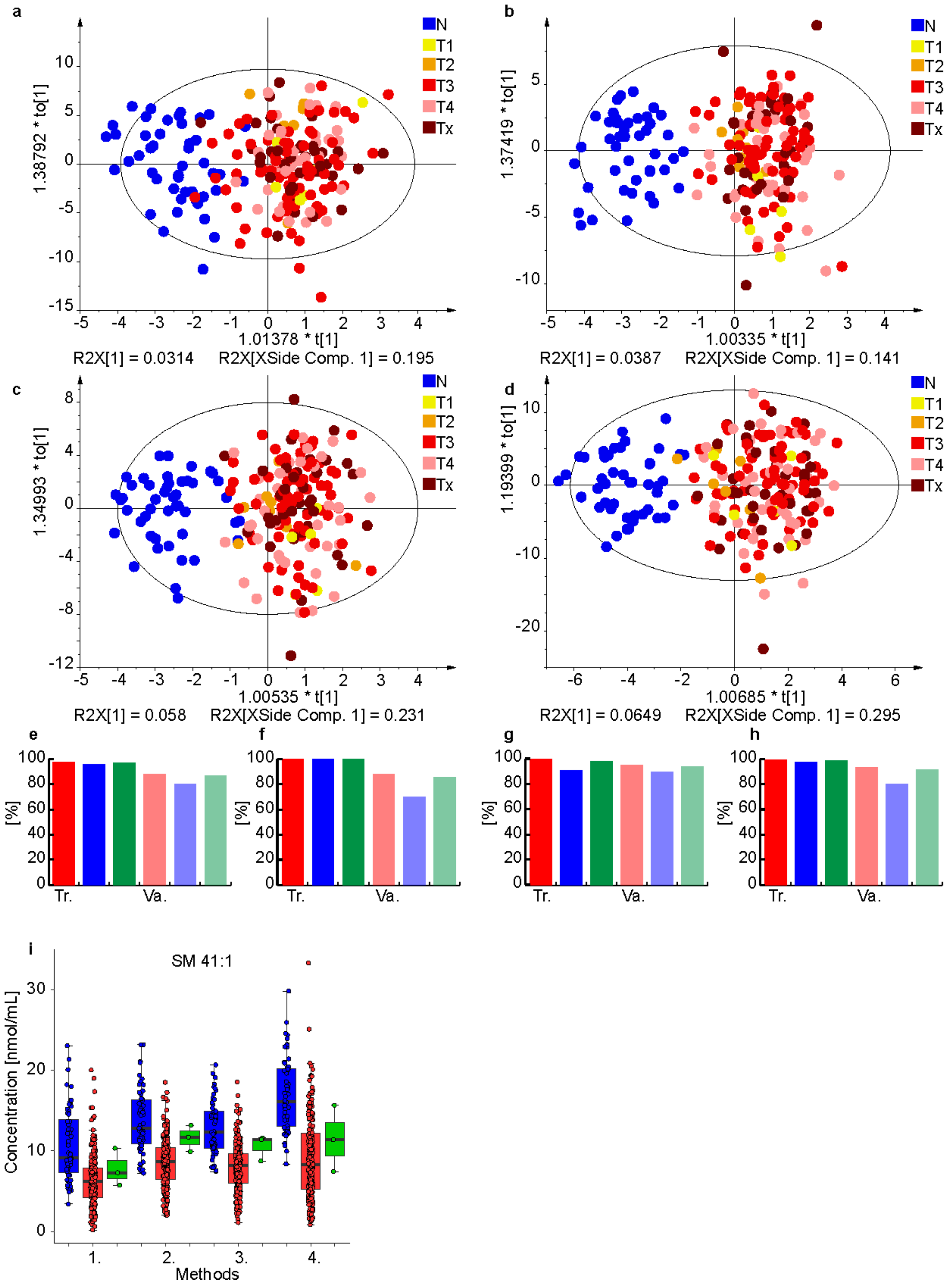

Supplementary Fig. 3 Results for the lipidomic profiling of female serum samples from PDAC patients (T) and healthy controls (N) in the Phase II. OPLS-DA for molar

concentrations of lipid species obtained for the training set: **a** UHPSFC/MS (161 T + 46 N), **b** shotgun MS (LR) (160 T + 46 N), **c** shotgun MS (HR) (161 T + 46 N), and **d** RP-UHPLC/MS (161 T + 46 N). Individual samples are colored according to their tumor (T) stage: T1 - yellow, T2 - orange, T3 - red, T4 - rose, and Tx - brown (information about the stage is not available). Sensitivity (red), specificity (blue), and accuracy (green) values in percentage for the training and validation sets: **e** UHPSFC/MS, **f** shotgun MS (LR), **g** shotgun MS (HR), and **h** RP-UHPLC/MS. **i** Box plots of molar lipid concentrations normalized with the NIST reference material determined in PDAC patients (221 T), controls (56 N), and pancreatitis (3 Pan) patients including both validation and training sets for SM 41:1 using UHPSFC/MS (Method 1), shotgun MS (LR) (Method 2), shotgun MS (HR) (Method 3), and RP-UHPLC/MS (Method 4). In each box plot, the centerline represents the median, the bounds represent the 1st and 3rd quartile and whiskers span 1.5 fold inter-quartile range from the median.

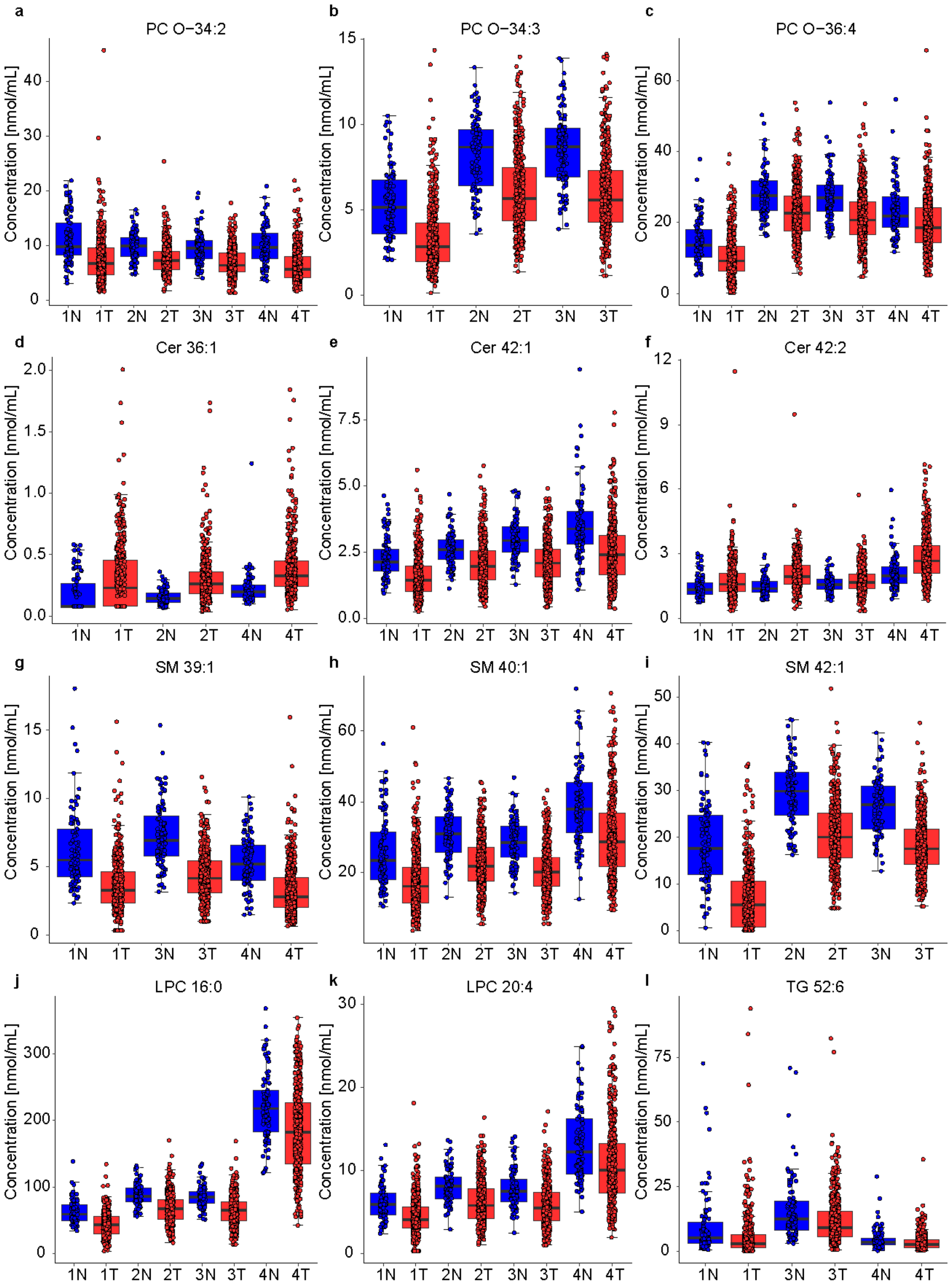

Supplementary Fig. 4 Selected box plots for the Phase II. Lipid concentrations normalized with the NIST reference material determined in PDAC patients (443 T) and healthy controls

(95 N) including both validation and training sets and both genders: **a** PC O-34:2, **b** PC O-34:3, **c** PC O-36:4, **d** Cer 36:1, **e** Cer 42:1, **f** Cer 42:2, **g** SM 39:1, **h** SM 40:1, **i** SM 42:1, **j** LPC 16:0, **k** LPC 20:4, and **l** TG 52:6 for UHPSFC/MS (Method 1), shotgun MS (LR) (Method 2), shotgun MS (HR) (Method 3), and RP-UHPLC/MS (Method 4). In each box plot, the centerline represents the median, the bounds represent the 1st and 3rd quartile and whiskers span 1.5 fold inter-quartile range from the median.

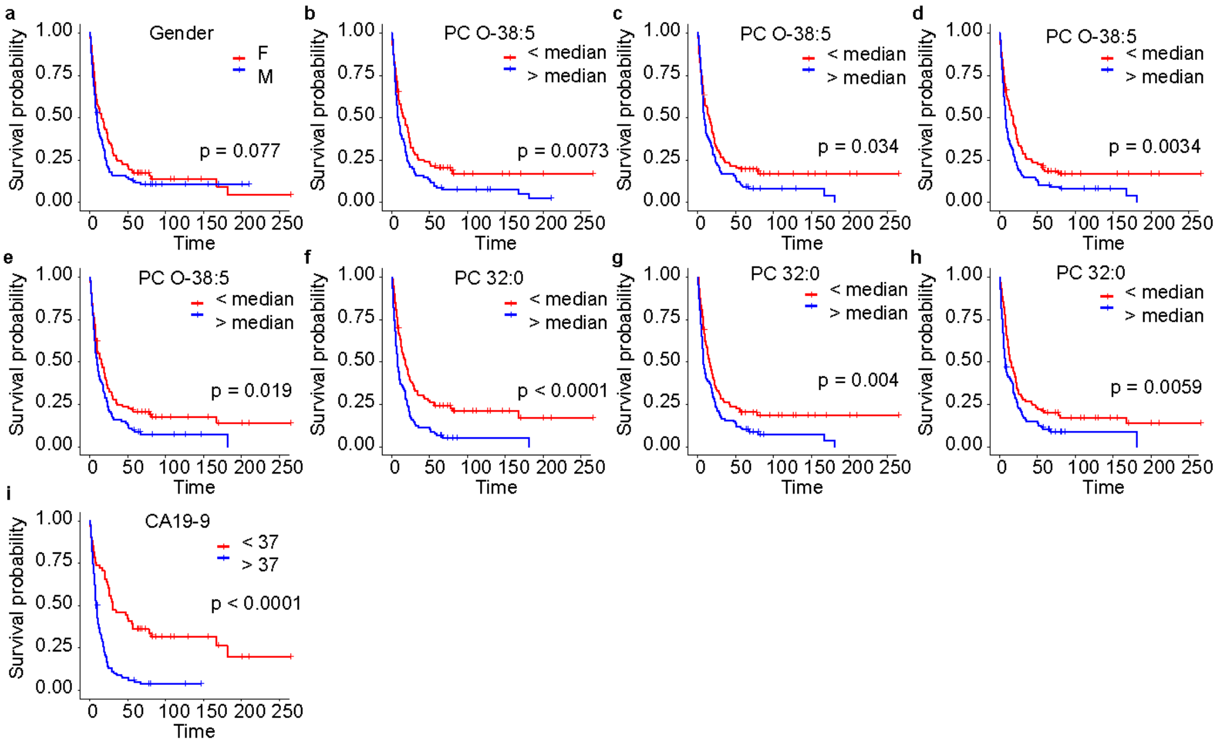

Supplementary Fig. 5 Supplementary Fig. 5 Potential of selected dysregulated lipid species for the survival prognosis in the Phase II using Kaplan-Meier plots together with the two sided log rank test p-values**. a** Gender (102 males and 98 females). **b** PC O-38:5 measured by UHPSFC/MS (n=98 for binary code 0, and n=102 for binary code 1). **c** PC O-38:5 measured by shotgun MS (LR) (n=103 for 0, and n=97 for 1). **d** PC O-38:5 measured by shotgun MS (HR) (n=103 for 0, and n=97 for 1). **e** PC O-38:5 measured by RP-UHPLC/MS (n=98 for 0, and n=102 for 1). **f** PC 32:0 measured by shotgun MS (LR) (n=94 for 0, and n=106 for 1). **g** PC 32:0 measured by shotgun MS (HR) (n=91 for 0, and n=109 for 1). **h** PC 32:0 measured by RP-UHPLC/MS (n=90 for 0, and n=110 for 1). **i** CA 19-9 (n=62 for 0, and n=138 for 1).

Supplementary Table 1. Concentrations of the internal standard (IS) mixture used for the lipidomic quantitation in Phase I (discovery) for UHPSFC/MS, shotgun MS, and MALDI-MS in lab 1.

IS	Stock concentration [µg/µL]	Volume for UHPSFC/MS and MALDI-MS [µL]	Volume for shotgun MS [µL]	Concentration in serum	
				UHPSFC/MS and MALDI-MS [nmol/mL]	Shotgun MS [nmol/mL]
CE 16:0 D7	2	71.4	85.0	125.0	125.0
Cer 30:1	2	3.0	2.5	6.9	4.8
DG 24:2	2	71.4	5.0	174.5	10.3
Hex2Cer 30:1	2	-	5.0	-	5.8
HexCer 30:1	2.1	-	5.0	-	7.6
Chol D7	2	142.8	170.0	401.0	401.0
LPC 17:0	2.1	12.6	15.0	28.7	28.7
LPE 14:0	2	4.2	5.0	10.9	10.9
LPG 14:0[a]	2.1	-	10.0	-	20.4
MG 19:1	2	40.7	10.0	121.6	25.1
PA 28:0	2	-	5.0	0.0	7.6
PC 28:0	2	21.0	25.0	34.3	34.3
PE 28:0	2	2.5	5.0	4.4	7.3
PG 28:0[a]	2	-	2.5	-	3.4
PS 28:0[a]	2	-	5.0	-	6.6
SM 30:1	2	12.6	15.0	21.5	21.5
SHexCer 30:1[b]	0.25	4.2	2.5	0.8	0.4
TG 57:3	2	12.6	170.0	15.0	170.3
Solvent	$CHCl_3$:IPA (2:8, v/v)	867.2	632.5		
Total volume		1266.3	1507.5		

Supplementary Table 2. Concentrations of the internal standard (IS) mixture used for the lipidomic quantitation in Phase II (qualification) and Phase III (verification) for UHPSFC/MS in lab 1.

IS	Stock concentration [µg/µL]	Volume [µL]	IS Mix [µg/mL]	Concentration in serum	
				[µg/mL]	[nmol/mL]
CE 16:0 D7	6	225	270.0	216.0	342.0
Cer 30:1	2	4	1.6	1.3	2.7
Cer 35:1	2	4	1.6	1.3	2.3
Cer C18 D7	1	8	1.6	1.3	2.2
DG 24:2	2	16	6.4	5.1	11.3
DG 36:2 D5	1	22.5	4.5	3.6	5.8
DG 33:1 D7	1	21	4.2	3.4	5.7
Chol D7	6	95	114.0	91.2	231.6
LPC 17:0	2	55	22.0	17.6	34.6
LPC 18:1 D7	1	115	23.0	18.4	34.8
MG 19:1	4	7	5.6	4.5	12.1
MG 18:1 D7	1	26	5.2	4.2	11.4
PC 33:1 D7	1	500	100.0	80.0	106.2
PC 28:0	4	120	96.0	76.8	113.4
PC 44:2	2	160	64.0	51.2	57.0
PC 44:0	2	270	108.0	86.4	95.8
SM 30:1	2	70	28.0	22.4	34.6
SM 36:2 D9	1	160	32.0	25.6	34.7
TG 48:1 D7	1	290	58.0	46.4	57.1
TG 57:3	6	55	66.0	52.8	57.0
Solvent	$CHCl_3$:MeOH (1:1, v/v)	2776.5			
Total volume		5000			

Supplementary Table 3. Concentrations of the internal standard (IS) mixture used for the lipidomic quantitation in Phase II (qualification) for shotgun MS (LR and HR) in lab 2.

IS	Stock concentration [µg/µL]	Volume [µL]	IS Mix[µg/mL]	Concentration in serum	
				[µg/mL]	[nmol/mL]
PC 28:0	1	1750	35	87.5	129.2
PC 44:0	1	2500	50	125.0	138.6
LPC 13:0	1	750	15	37.5	82.7
LPC 19:0	1	750	15	37.5	69.8
LPE 13:0	1	50	1	2.5	6.1
Cer 17:0	0.1	250	0.5	1.3	2.3
Cer 14:0	0.1	250	0.5	1.3	2.5
PE 28:0	1	200	4	10.0	15.7
PE 40:0	1	200	4	10.0	12.4
PS 28:0	1	150	3	7.5	11.0
PS 40:0	1	150	3	7.5	8.8
PI 34:0	0.1	2500	5	12.5	14.9
CE 17:0	10	500	100	250.0	391.5
CE 22:0	10	500	100	250.0	352.8
Chol D7	10	750	150	375.0	952.7
DG 28:0	1	600	12	30.0	58.5
DG 40:0	1	600	12	30.0	44.1
TG 51:0	2.5	1000	50	125.0	147.3
TG 57:0	1	2500	50	125.0	134.0
SM 30:1;2	1	500	10	25.0	38.7
HexCer 30:1;2	0.1	400	0.8	2.0	3.1
HexCer 35:1;2	0.1	400	0.8	2.0	2.8
Solvent	CHCl3:MeOH (9:1, v/v)	32750			
Total volume		50000			

Supplementary Table 4. Concentrations of the internal standard (IS) mixture used for the lipidomic quantitation in Phase II (qualification) for RP-UHPLC/MS in lab 3.

IS	Stock concentration [µg/µL]	Volume [µL]	IS Mix [µg/mL]	Concentration in serum	
				[µg/mL]	[nmol/mL]
Acylcarnitine 16:0 D3	0.08	47.5	0.03	0.5	1.2
Cardiolipin 14:0[b]	0.51	95.0	0.3	6.1	4.7
CE 18:0 D6	1.32	19000.0	164.8	3131.0	4750.0
Chol D7	7.87	190.0	9.8	187.0	475.0
DG 15:0_15:0	0.43	95.0	0.3	5.1	9.5
dhCer(18:0/8:0)	0.09	95.0	0.1	1.0	2.4
Cer(m18:1/12:0)	0.09	95.0	0.1	1.1	2.4
TG 17:0_17:0_17:0	0.68	47.5	0.2	4.0	4.7
TG 12:0_12:0_12:0	0.26	201.4	0.3	6.4	10.1
LPE 14:0	0.34	47.5	0.1	2.0	4.7
LPC 13:0	0.36	47.5	0.1	2.2	4.7
PC 13:0_13:0	0.32	76.0	0.2	3.1	4.7
PE 17:0_17:0	0.29	47.5	0.1	1.7	2.4
PG 17:0_17:0	0.30	47.5	0.1	1.8	2.4
PS 17:0_17:0	0.31	23.9	0.05	0.9	1.2
PI 12:0_13:0[b]	0.01	4306.0	0.3	5.5	7.6
S1P (d18:1) $^{13}C_2D_2$	0.10	29.7	0.02	0.4	1.0
SPH/Cer 1-mix[c]		4000.0			
Sphingosine (d17:1)[c]	0.01		0.2	3.5	12.1
Sphinganine (d17:0)[c]	0.01		0.2	3.5	12.3
S1P(d17:1)c	0.01		0.2	4.6	12.7
S1P (d17:0)[c]	0.01		0.2	4.6	12.5
Hex2Cer (d18:1/12:0)[c]	0.02		0.5	10.0	12.3
HexCer (d18:1/12:0)[c]	0.02		0.4	7.8	12.1
SM (d18:1/12:0)[c]	0.02		0.4	7.9	12.1
Cer (d18:1/12:0)[c]	0.01		0.3	5.8	12.1
C1P (d18:1/12:0)[c]	0.01		0.4	6.7	11.9
Cer (d18:1/25:0)[c]	0.02		0.4	8.1	12.2
Solvent	BuOH:MeOH (1:1, v/v)	123508			
Total volume		152000			

[a] IS is the sodium salt; [b] IS is the ammonium salt; [c] IS spiked in as part of an pre-mixed commercial mixture

Supplementary Table 5. Summary table of significant lipid species according to the log rank test p-value observed for multiple methods in Phase II (qualification) applying the Kaplan-Meier Survival analysis.

Lipid species	Note (detected and significant/methods)	Survival probability higher when
**PC 32:0**	4/4	0
**PC O-38:5**	4/4	0
**PI 34:1**	3/3	1
**PI 36:3**	3/3	1
**LPE 18:1**	2/2	1
**LPE 18:2**	2/2	1
**Cer 36:1**	3/4	0
**Cer 38:1**	3/4	0
**Cer 42:2**	3/4	0
**LPC 18:2**	3/4	1
**SM 42:2**	3/4	0
**PI 32:0**	2/3	1
**PI 34:2**	2/3	1
**PI 36:1**	2/3	1
**PI 36:2**	2/3	1
**PI 38:3**	2/3	1
**PI 38:5**	2/3	1
**PE 40:6**	2/3	1
**PE O-36:3**	2/3	1
**PE O-36:4**	2/3	1
**CE 20:5**	2/3	1
**TG 50:4**	2/3	1
**TG 50:5**	2/3	1
**TG 52:6**	2/3	1
**TG 56:8**	2/3	0
**Cer 34:1**	2/4	0
**Cer 36:1**	2/4	0
**LPC 14:0**	2/4	1
**LPC 18:3**	2/4	1
**LPC 20:0**	2/4	1
**PC 40:6**	2/4	0
**PC O-40:5**	2/4	0
**PC O-42:4**	2/4	0
**SM 39:1**	2/4	1
**SM 41:1**	2/4	1
**SM 44:3**	2/4	1

Supplementary Table 6. Sensitivity, specificity, and accuracy values for training and validation set of Phase III and the comparison to literature values (Cohen et al.). Lipidomics and the combination of lipidomics with CA19-9 used the predictive response values obtained from the OPLS-DA models for the training set to calculate parameters. For CA19-9 the threshold was set according to the assay, values >37 U/mL were classified as cancer samples. The same threshold was used for the CA19-9 literature values from Cohen et al, to determine the prediction performance. The CancerSeek Test results (positive, negative) were used to compare to literature.

		Phase III (verification)								
		Lipidomics UHPSFC/MS			CA19-9			Lipidomics + CA19_9 UHPSFC/MS		
		Male	Female	Both	Male	Female	Both	Male	Female	Both
Training set	Specificity	94.3	94.4	91.9	98.4	96.0	97.2	99.2	98.4	95.1
	Sensitivity	98.6	94.3	95.3	66.1	66.2	66.1	97.7	94.8	96.0
	T1	100.0	100.0	92.9	33.3	37.5	35.7	100.0	100.0	100.0
	T2	100.0	86.7	95.8	55.6	20.0	33.3	88.9	86.7	87.5
	T3	98.8	92.8	95.9	62.4	53.0	57.7	97.7	91.6	96.4
	T4	100.0	97.0	96.7	70.9	89.2	80.8	100.0	98.5	97.5
	Tx	96.8	94.9	93.1	71.4	79.5	74.5	96.8	97.4	95.1
	Accuracy	97.1	94.3	94.1	77.6	77.2	77.4	98.2	96.1	95.7
Validation set	Specificity	83.3	80.0	87.5	100.0	100.0	100.0	100.0	100.0	93.8
	Sensitivity	94.6	80.0	87.9	71.4	56.7	63.8	94.6	78.3	87.1
	T1	100.0	33.3	60.0	100.0	33.3	60.0	100.0	33.3	60.0
	T2	100.0	50.0	72.7	57.1	0.0	36.4	100.0	25.0	63.6
	T3	89.5	94.4	91.9	63.2	50.0	56.8	89.5	100.0	91.9
	T4	100.0	88.9	95.7	78.6	88.9	82.6	92.9	88.9	95.7
	Tx	92.9	76.9	87.5	78.6	61.5	67.5	100.0	73.1	87.5
	Accuracy	93.5	80.0	87.9	74.2	62.9	68.2	95.2	81.4	87.9

		Literature Cancer Seek					
		Cancer Seek test ctDNA+proteins			CA19-9 only CA19-9		
		Male	Female	Both	Male	Female	Both
Training set	Specificity	99.1	99.2	99.1	97.7	97.1	97.4
	Sensitivity	72.7	71.1	72.0	74.5	76.3	75.3
	I	25.0	-	25.0	0.0	-	0.0
	II	76.6	69.4	73.5	78.7	75.0	77.1
	II	75.0	100.0	83.3	100.0	100.0	100.0
	Accuracy	96.1	96.6	96.4	95.1	95.2	95.1

Supplementary Table 7. Summary of predictive components and orthogonal in X components, needed to reproduce the OPLS-DA models in SIMCA.

Predictive + Orthogonal in X components				
Phase	Method	Male	Female	Lab
Phase I (discovery)	UHPSFC/MS	1+4+0	1+5+0	lab 1
	Shotgun MS	1+4+0	1+4+0	lab 1
	MALDI-MS	1+3+0	1+2+0	lab 1
Phase II (qualification)	UHPSFC/MS	1+5+0	1+4+0	lab 1
	Shotgun MS (LR)	1+1+0	1+5+0	lab 2
	Shotgun MS (HR)	1+5+0	1+5+0	lab 2
	RP-UHPLC/MS	1+8+0	1+4+0	lab 3
Phase III (verification)	UHPSFC/MS	1+6+0	1+5+0	lab 1
Normalized concentrations				
Phase II (qualification)	UHPSFC/MS	1+4+0	1+6+0	lab 1
	Shotgun MS (LR)	1+1+0	1+2+0	lab 2
	Shotgun MS (HR)	1+9+0	1+6+0	lab 2
	RP-UHPLC/MS	1+9+0	1+4+0	lab 3
Phase III (verification)	UHPSFC/MS	1+6+0	1+5+0	lab 1
