# Supplementary material for: Lipidomic profiling of human serum enables detection of pancreatic cancer
Source: Nat Commun. 2022 Jan 10;13:124. doi: 10.1038/s41467-021-27765-9 (PMC8748654; doi:10.1038/s41467-021-27765-9)
Supplement: Supplementary file 3 — Description of Additional Supplementary Files [file 41467_2021_27765_MOESM3_ESM.pdf]

Description of Additional Supplementary Files

Supplementary Data 1-20: Contains clinical information of training and validation sample sets. The lipidomic data of discovery, qualification, and verification phases, the statistical evaluation results for the differentiation of patient and control samples. Results of the Kaplan-Meier survival analysis.

Supplementary Data 1. Overview of studied human subjects in this study.

Supplementary Data 2. Clinical information for all samples used in this study.

Supplementary Data 3. Lipid shorthand nomenclature used throughout this work.

Supplementary Data 4. Molar concentrations [nmol/mL] of individual lipids measured in Phase I (discovery) together with the statistical evaluation using UHPSFC/MS.

Supplementary Data 5. Molar concentrations [nmol/mL] of individual lipids measured in Phase I (discovery) together with the statistical evaluation using **s**hotgun MS.

Supplementary Data 6. Molar concentrations [nmol/mL] of individual lipids measured in Phase I (discovery) together with the statistical evaluation using MALDI-MS.

Supplementary Data 7. Molar concentrations [nmol/mL] of individual lipids measured in Phase II (qualification) together with the statistical evaluation and normalized concentrations (located below the summary table of the statistical evaluation) using UHPSFC/MS. (N: control sample; T: PDAC cancer sample; Pan: pancreatitis).

Supplementary Data 8. Molar concentrations [nmol/mL] of individual lipids measured in Phase II (qualification) together with the statistical evaluation and normalized concentrations (located below the summary table of the statistical evaluation) using **s**hotgun MS (LR).

Supplementary Data 9. Molar concentrations [nmol/mL] of individual lipids measured in Phase II (qualification) together with the statistical evaluation and normalized concentrations (located below the summary table of the statistical evaluation) using **s**hotgun MS (HR).

Supplementary Data 10. Molar concentrations [nmol/mL] of individual lipids measured in Phase II (qualification) together with the statistical evaluation using RP-UHPLC/MS.

Supplementary Data 11. Molar concentrations [nmol/mL] of individual lipids measured in Phase II (qualification) together with the statistical evaluation and normalized concentrations (located below the summary table of the statistical evaluation) using RP-UHPLC/MS - lipid fatty acyl level were transformed to lipid species level.

Supplementary Data 12. Molar concentrations [nmol/mL] of individual lipids measured by UHPSFC/MS in Phase III (verification) together with the statistical evaluation and normalized concentrations (located below the summary table of the statistical evaluation).

Supplementary Data 13. Sensitivity, specificity, and accuracy values for training and validation sets. Calculated from the predictive response values from the OPLS-DA models for the training set.

Supplementary Data 14. Statistical parameters calculated from molar concentrations for the training set for all methods and all lipids with fold change ≥20%, p-value<0.05, and VIP value >1 for Phase I (discovery).

Supplementary Data 15. Statistical parameters calculated from molar concentrations for the training set for all methods and all lipids with fold change ≥20%, p-value<0.05, and VIP value >1 for Phase II (qualification).

Supplementary Data 16. Statistical parameters calculated from molar concentrations for the training set for all methods and all lipids with fold change ≥20%, p-value<0.05, and VIP value >1 for Phase III (verification).

Supplementary Data 17. Predicted response values calculated from OPLS-DA models for all methods, all phases, and validation sample set.

Supplementary Data 18. Predicted response values calculated from OPLS-DA models for all methods, all phases, and training sample set.

Supplementary Data 19. Survival analysis data from Kaplan-Meier plots for individual methods in Phase II (qualification). Lipid species concentrations normalized to the NIST reference material, where values lower than median are classified as 0, and values higher than median are 1 for all lipids with a two-sided log rank p-value <0.05.

Supplementary Data 20. Multiple reaction monitoring (MRM) settings for RP-UHPLC/MS measurements in lab 3.
