# Supplementary material for: Lipidomic profiling of human serum enables detection of pancreatic cancer
Source: Nat Commun. 2022 Jan 10;13:124. doi: 10.1038/s41467-021-27765-9 (PMC8748654; doi:10.1038/s41467-021-27765-9)
Supplement: Supplementary file 5 — Reporting Summary [file 41467_2021_27765_MOESM5_ESM.pdf]

nature portfolio

Corresponding author(s): Michal Holčapek

Last updated by author(s): November 21st, 2021

Reporting Summary

Nature Portfolio wishes to improve the reproducibility of the work that we publish. This form provides structure for consistency and transparency in reporting. For further information on Nature Portfolio policies, see our Editorial Policies and the Editorial Policy Checklist.

Statistics

For all statistical analyses, confirm that the following items are present in the figure legend, table legend, main text, or Methods section.

n/a	Confirmed	
☐	☒	The exact sample size (*n*) for each experimental group/condition, given as a discrete number and unit of measurement
☐	☒	A statement on whether measurements were taken from distinct samples or whether the same sample was measured repeatedly
☐	☒	The statistical test(s) used AND whether they are one- or two-sided<br>*Only common tests should be described solely by name; describe more complex techniques in the Methods section.*
☐	☒	A description of all covariates tested
☐	☒	A description of any assumptions or corrections, such as tests of normality and adjustment for multiple comparisons
☐	☒	A full description of the statistical parameters including central tendency (e.g. means) or other basic estimates (e.g. regression coefficient) AND variation (e.g. standard deviation) or associated estimates of uncertainty (e.g. confidence intervals)
☐	☒	For null hypothesis testing, the test statistic (e.g. *F*, *t*, *r*) with confidence intervals, effect sizes, degrees of freedom and *P* value noted<br>*Give P values as exact values whenever suitable.*
☒	☐	For Bayesian analysis, information on the choice of priors and Markov chain Monte Carlo settings
☒	☐	For hierarchical and complex designs, identification of the appropriate level for tests and full reporting of outcomes
☒	☐	Estimates of effect sizes (e.g. Cohen's *d*, Pearson's *r*), indicating how they were calculated

Our web collection on statistics for biologists contains articles on many of the points above.

Software and code

Policy information about availability of computer code

Data collection	The vendor software packages for the data collection, which were mentioned in the manuscript (with appropriate versions): for SFC (lab 1 – Czech Republic): the MassLynx (version 4.1 – Czech Republic), for shotgun (lab 1): the AB Sciex Analyst software (version 1.6.2), for RP (lab 3 – Singapore): the MassHunter Acquisition Software (version B.09.00 – B9037.0).<br>Data were subsequently processed via the following software: for SFC (lab 1 – Czech Republic) MarkerLynx (version 4.1), Waters compression tool (version 4.1), and LipidQuant (version 1.0), for shotgun (lab 1 – Czech Republic) LipidView (version 1.2) and LipidQuant (version 1.0), for shotgun (lab 2 – Germany) – ALEX software, including: extractor, converter and target list generator (http://mslipidomics.info/contents/?page_id=133) and Microsoft Excel 2016 (version 16.0.5239.1001), for RP (lab 3 – Singapore) Agilent Mass Hunter Quantitative Analysis software for QQQ (version B.10.00), Microsoft Excel (version 1808) and R (version 4.0.0) and the following packages: here (version 0.1), dplyr (version 0.8.5), tidyr (version 1.0.3), purrr (version 0.3.4), readr (version 1.3.1), lubridate (version 1.7.8), stringr (version 1.4.0).
Data analysis	For the data analysis and visualization, the following software and analytical platforms were used: Microsoft Excel Professional Plus 2016 (version 16.0.5239), SIMCA (version 13.0.3), Cytoscape software (version 3.8.0), Metaboanalyst platform (version 4.0), Adobe Illustrator CC 2018 (version 22.1, 64 bit), R (version 3.6.2) and packages: ggplot2 (version 3.3.3), ggpubr (version 0.4.0), rstatix (version 0.6.0), AUC (version 0.3.0), survival (version 3.2-7), survminer (version 0.4.8), readxl (version 1.3.1), dplyr (version 1.0.2).

For manuscripts utilizing custom algorithms or software that are central to the research but not yet described in published literature, software must be made available to editors and reviewers. We strongly encourage code deposition in a community repository (e.g. GitHub). See the Nature Portfolio guidelines for submitting code & software for further information.

March 2021

Data

Policy information about availability of data

All manuscripts must include a data availability statement. This statement should provide the following information, where applicable:

- Accession codes, unique identifiers, or web links for publicly available datasets
- A description of any restrictions on data availability
- For clinical datasets or third party data, please ensure that the statement adheres to our policy

Data to support and reproduce our findings are available in the manuscript, in the supplementary information, the supplementary data, the source data, the supplementary source data and on-line (Figshare).
Raw data, instructions for the software handling and the software are deposited at figshare.com (see Methods section of the manuscript) and on the homepage of the corresponding author-https://holcapek.upce.cz/

Field-specific reporting

Please select the one below that is the best fit for your research. If you are not sure, read the appropriate sections before making your selection.

☒ Life sciences ☐ Behavioural & social sciences ☐ Ecological, evolutionary & environmental sciences

For a reference copy of the document with all sections, see nature.com/documents/nr-reporting-summary-flat.pdf

Life sciences study design

All studies must disclose on these points even when the disclosure is negative.

Sample size	Sample size was obtained based on the serum samples availability for the study presented here. No specific statistical approaches were applied to predetermine the sample size.<br>The case/control type of study was applied, including three phases: (1) discovery Phase I (262T + 102N), (2) qualification Phase II (444T + 98N + 12 pancreatitis) (3) verification Phase III (546T + 262N + 22 pancreatitis). In total, 830 samples from four different collection sites. Within them, we managed to collect 24 males and 30 females T1/T2 PDAC samples. It must be highlighted that early-stage PDAC patients typically account for a small subgroup among all cases.<br>Sample sizes were sufficient to build statistical models demonstrating the feasibility of lipid profiling for PDAC detection. Even though 22 samples were collected from patients with pancreatitis, we found no clear influence of the disease on lipids' concentrations, having essential importance for the obtained statistical models; similarly in the diabetes mellitus case.
Data exclusions	No clinical exclusion criteria were applied. Quality control procedures allowed the detection of outliers resulting from sample preparation or instrumental reasons. Outliers detected based on the Principal Component Analysis were also excluded from further statistical analysis, i.e. observations far from the centroid of the elliptical region, which usually encompasses most of the observations (the 95% confidence limit for Hotelling's T^2) - as described in the Methods section of the manuscript. In Phase I, sample No. 355 was excluded from the UHPSFC/MS data set, and sample No. 210 for the shotgun MS data set. In Phase II, samples No. 246 and 500 were excluded from the low resolution shotgun MS data set, and samples No. 246 and 409 from the high resolution shotgun MS data set.
Replication	Please see Phase I description in the main text. The samples were independently worked up and measured by various methods. Please see the Phase II description in the main text and methods section to the manuscript, i.e. the qualification step. The Phase II experiments were performed by 3 independent laboratories, using 4 different analytical methods, using one set containing 554 samples. he samples were independently worked up and measured by various methods. Furthermore, please see Phase III description (the final verification step), measured again by the main method in the laboratory no. 1, using 830 samples, in total. No additional replication, than the independnet sample preparation work up and measurements by various methods and laboratories in Phase 1 and Phase II was performed.
Randomization	All samples within the particular phase were processed and measured in the randomized order (Kutools in Microsoft Excel). The stratified randomization based on the sample group, age, gender, and BMI was applied was used in the lab no. 3.
Blinding	At the sample preparation steps and measurements, operators had no information about the sample classification. The sample sets in all phases were divided into the training and validation sets. The sample classification for the training set was disclosed for the multivariate data analysis (MDA). The classification of the validation set was disclosed after the final prediction of the validation set. No fully blinded study was performed, with the external third side involved.

Reporting for specific materials, systems and methods

We require information from authors about some types of materials, experimental systems and methods used in many studies. Here, indicate whether each material, system or method listed is relevant to your study. If you are not sure if a list item applies to your research, read the appropriate section before selecting a response.

Materials & experimental systems

n/a	Involved in the study
☒	☐ Antibodies
☒	☐ Eukaryotic cell lines
☒	☐ Palaeontology and archaeology
☒	☐ Animals and other organisms
☐	☒ Human research participants
☒	☐ Clinical data
☒	☐ Dual use research of concern

Methods

n/a	Involved in the study
☒	☐ ChIP-seq
☒	☐ Flow cytometry
☒	☐ MRI-based neuroimaging

Human research participants

Policy information about studies involving human research participants

Population characteristics

All samples were collected in the Czech Republic, and 4 different collection sites can be distinguished. For more details, please see the Methods section - subsection: Serum samples. To see patients and healthy controls characteristics - please see Supplementary Table no. 1 and 2.

Recruitment

The study does not involve any specific participant recruitment strategy. It is a case/control type of study and it was performed using available samples, including cases/controls collected by the Institutions listed in the Method section and the Result section in the chapter Clinical samples. It is unlikely that the sample collection step had any impact on the findings described here.

Ethics oversight

Samples and ethical approval were provided by the following institutions: BBM of Masaryk Memorial Cancer Institute in Brno (554 samples, see Phase II), the First and Third Faculty of Medicine at the Charles University in Prague (147 samples), the University Hospital in Pilsen (31 samples) and by the Palacký University and University Hospital in Olomouc (98 samples).

Note that full information on the approval of the study protocol must also be provided in the manuscript.
